# Supplementary material for: Exposure Measurement Error Correction in Longitudinal Studies With Discrete Outcomes
Source: Stat Med. 2025 Jul 18;44(15-17):e70191. doi: 10.1002/sim.70191 (PMC12274082; doi:10.1002/sim.70191)
Supplement: Supplementary file 1 — Data S1. Supporting Information. [file SIM-44-0-s001.zip › sim_70191-sup-0003-Supinfo.pdf]

Supplementary Material: Exposure measurement error correction in longitudinal studies with discrete outcomes

Ce Yang, Ning Zhang, Jiaxuan Li, Unnati V. Mehta, Jaime E. Hart, Donna Spiegelman, Molin Wang

Department of Epidemiology, Harvard T.H. Chan School of Public Health, Boston, MA, USA

Department of Epidemiology, University of North Carolina, Chapel Hill, NC, USA

Department of Epidemiology, Harvard T.H. Chan School of Public Health, Boston, MA, USA

Department of Environmental Health, Harvard T.H. Chan School of Public Health, Boston, MA, USA

Department of Environmental Health, Harvard T.H. Chan School of Public Health, Channing Division of Network Medicine, Department of Medicine, Brigham and Women's Hospital, Harvard Medical School, Boston, MA, USA

Department of Biostatistics, Yale School of Public Health, New Haven, CT, USA

Departments of Epidemiology and Biostatistics, Harvard T.H. Chan School of Public Health, Channing Division of Network Medicine, Department of Medicine, Brigham and Women's Hospital and Harvard Medical School, Boston, MA, USA.

Corresponding Author: Molin Wang.

Appendix A. Derivation of the variance estimates of the proposed estimators

The variance of the resulting estimator has the sandwich form following the theory of unbiased estimating equations [Liang and Zeger, 1986],

$$\begin{aligned}\mathrm{Var}(\hat{\boldsymbol{\theta}}) &\approx E\left(\frac{\partial \boldsymbol{\psi}}{\partial \boldsymbol{\theta}}\right)^{-1} \mathrm{Var}(\boldsymbol{\psi}(\boldsymbol{\theta})) E\left(\frac{\partial \boldsymbol{\psi}}{\partial \boldsymbol{\theta}}\right)^{-1'} \\ &= \mathbf{B}\left(\boldsymbol{\theta}\right)^{-1} \mathbf{A}\left(\boldsymbol{\theta}\right) \mathbf{B}\left(\boldsymbol{\theta}\right)^{-1'}.\end{aligned}$$

We present estimates of $\mathbf{A}\left(\boldsymbol{\theta}\right)$ and $\mathbf{B}\left(\boldsymbol{\theta}\right)$ in the next two subsections.

A1. Single measurement in the validation study

$\mathbf{A}\left(\boldsymbol{\theta}\right)$ can be estimated by:

$$\widehat{\mathbf{A}}\left(\boldsymbol{\theta}\right) = \widehat{\mathrm{Var}}(\boldsymbol{\psi}) = \begin{pmatrix} \widehat{\mathrm{Var}}(\boldsymbol{\psi}_{\boldsymbol{\alpha}}) & \widehat{\mathrm{Cov}}(\boldsymbol{\psi}_{\boldsymbol{\alpha}}, \boldsymbol{\psi}_{\boldsymbol{\beta}}) \\ \widehat{\mathrm{Cov}}(\boldsymbol{\psi}_{\boldsymbol{\beta}}, \boldsymbol{\psi}_{\boldsymbol{\alpha}}) & \widehat{\mathrm{Var}}(\boldsymbol{\psi}_{\boldsymbol{\beta}}) \end{pmatrix} |_{\boldsymbol{\alpha}=\hat{\boldsymbol{\alpha}}, \boldsymbol{\beta}=\hat{\boldsymbol{\beta}}},$$

where

$$\widehat{\mathrm{Var}}(\boldsymbol{\psi}_{\boldsymbol{\alpha}}) = \sum_{i=n_1+1}^{n_1+n_2} \mathbf{C}_{i1}\mathbf{C}_{i1}' \left(c_i - \mathbf{C}_{i1}'\boldsymbol{\alpha}\right)^2 |_{\boldsymbol{\alpha}=\hat{\boldsymbol{\alpha}}}.$$

For an external validation study (EVS), $\widehat{\mathbf{A}}\left(\boldsymbol{\theta}\right)$ is block diagonal and

$$\widehat{\mathrm{Var}}(\boldsymbol{\psi}_{\boldsymbol{\beta}}) \approx \sum_{i=1}^{n_1} \left[\left(\frac{\partial \boldsymbol{\mu}_i}{\partial \boldsymbol{\beta}}\right)' \boldsymbol{\Sigma}_i^{-1}(\mathbf{Y}_i - \boldsymbol{\mu}_i)\right] \left[\left(\frac{\partial \boldsymbol{\mu}_i}{\partial \boldsymbol{\beta}}\right)' \boldsymbol{\Sigma}_i^{-1}(\mathbf{Y}_i - \boldsymbol{\mu}_i)\right]' |_{\boldsymbol{\mu}_i=\hat{\boldsymbol{\mu}}_i}.$$

For an internal validation study (IVS),

$$\widehat{\mathrm{Cov}}(\boldsymbol{\psi}_{\boldsymbol{\alpha}}, \boldsymbol{\psi}_{\boldsymbol{\beta}}) \approx \sum_{i=n_1+1}^{n_1+n_2} \mathbf{C}_{i1}\left(c_i - \mathbf{C}_{i1}'\boldsymbol{\alpha}\right) \left[\left(\frac{\partial \boldsymbol{\mu}_i}{\partial \boldsymbol{\beta}}\right)' \boldsymbol{\Sigma}_i^{-1}(\mathbf{Y}_i - \boldsymbol{\mu}_i)\right]' |_{\boldsymbol{\mu}_i=\hat{\boldsymbol{\mu}}_i, \boldsymbol{\alpha}=\hat{\boldsymbol{\alpha}}}$$

and

$$\widehat{\mathrm{Var}}(\boldsymbol{\psi}_{\boldsymbol{\beta}}) \approx \sum_{i=1}^{n_1+n_2} \left[\left(\frac{\partial \boldsymbol{\mu}_i}{\partial \boldsymbol{\beta}}\right)' \boldsymbol{\Sigma}_i^{-1}(\mathbf{Y}_i - \boldsymbol{\mu}_i)\right] \left[\left(\frac{\partial \boldsymbol{\mu}_i}{\partial \boldsymbol{\beta}}\right)' \boldsymbol{\Sigma}_i^{-1}(\mathbf{Y}_i - \boldsymbol{\mu}_i)\right]' |_{\boldsymbol{\mu}_i=\hat{\boldsymbol{\mu}}_i}.$$

$\mathbf{B}\left(\boldsymbol{\theta}\right)$ can be estimated by

$$\widehat{\mathbf{B}}\left(\boldsymbol{\theta}\right) = E\left(\frac{\partial \boldsymbol{\psi}}{\partial \boldsymbol{\theta}}\right) |_{\boldsymbol{\theta}=\hat{\boldsymbol{\theta}}} = E\begin{pmatrix} \frac{\partial \boldsymbol{\psi}_{\boldsymbol{\alpha}}}{\partial \boldsymbol{\alpha}} & \frac{\partial \boldsymbol{\psi}_{\boldsymbol{\alpha}}}{\partial \boldsymbol{\beta}} \\ \frac{\partial \boldsymbol{\psi}_{\boldsymbol{\beta}}}{\partial \boldsymbol{\alpha}} & \frac{\partial \boldsymbol{\psi}_{\boldsymbol{\beta}}}{\partial \boldsymbol{\beta}} \end{pmatrix} |_{\boldsymbol{\alpha}=\hat{\boldsymbol{\alpha}}, \boldsymbol{\beta}=\hat{\boldsymbol{\beta}}},$$

where

$$\begin{aligned} E\left(\frac{\partial \boldsymbol{\psi}_{\boldsymbol{\alpha}}}{\partial \boldsymbol{\alpha}}\right) &= -\sum_{i=n_1+1}^{n_1+n_2} \mathbf{C}_{i1}\mathbf{C}_{i1}'; \\ E\left(\frac{\partial \boldsymbol{\psi}_{\boldsymbol{\alpha}}}{\partial \boldsymbol{\beta}}\right) &= \mathbf{0}; \\ E\left(\frac{\partial \boldsymbol{\psi}_{\boldsymbol{\beta}}}{\partial \boldsymbol{\beta}}\right) &= -\sum_{i=1}^{N} \left(\frac{\partial \boldsymbol{\mu}_i}{\partial \boldsymbol{\beta}}\right)' \boldsymbol{\Sigma}_i^{-1} \frac{\partial \boldsymbol{\mu}_i}{\partial \boldsymbol{\beta}} |_{\boldsymbol{\mu}_i=\hat{\boldsymbol{\mu}}_i}; \end{aligned}$$

$$E\left(\frac{\partial \boldsymbol{\psi}_{\boldsymbol{\beta}}}{\partial \alpha_l}\right) = \sum_{i=1}^{N}\left[\left(\frac{\partial \boldsymbol{\mu}_i}{\partial \boldsymbol{\beta}\partial \alpha_l}\right)' \boldsymbol{\Sigma}_i^{-1}(\mathbf{Y}_i - \boldsymbol{\mu}_i) - \left(\frac{\partial \boldsymbol{\mu}_i}{\partial \boldsymbol{\beta}}\right)' \boldsymbol{\Sigma}_i^{-1}\frac{\partial \boldsymbol{\mu}_i}{\partial \alpha_l}\right]|_{\boldsymbol{\mu}_i=\hat{\boldsymbol{\mu}}_i}$$
$$= -\sum_{i=1}^{N}\left[\left(\frac{\partial \boldsymbol{\mu}_i}{\partial \boldsymbol{\beta}}\right)' \boldsymbol{\Sigma}_i^{-1}\frac{\partial \boldsymbol{\mu}_i}{\partial \alpha_l}\right]|_{\boldsymbol{\mu}_i=\hat{\boldsymbol{\mu}}_i}$$

for $l = 1, \dots, \dim(\boldsymbol{\alpha})$. Recall that $N = n_1 + n_2$ in the IVS and $N = n_1$ in the EVS. It follows that

$$\left(\frac{\partial \hat{\boldsymbol{\mu}}_i}{\partial \boldsymbol{\beta}}\right)' = \left(\frac{\partial g^{-1}\left(\hat{\mathbf{X}}_{i1}'\boldsymbol{\beta}\right)}{\partial \boldsymbol{\beta}}, \dots, \frac{\partial g^{-1}\left(\hat{\mathbf{X}}_{im_i}'\boldsymbol{\beta}\right)}{\partial \boldsymbol{\beta}}\right);$$
$$\left(\frac{\partial \hat{\boldsymbol{\mu}}_i}{\partial \boldsymbol{\beta}\partial \alpha_l}\right)' = \left(\frac{\partial g^{-1}\left(\hat{\mathbf{X}}_{i1}'\boldsymbol{\beta}\right)}{\partial \boldsymbol{\beta}\partial \alpha_l}, \dots, \frac{\partial g^{-1}\left(\hat{\mathbf{X}}_{im_i}'\boldsymbol{\beta}\right)}{\partial \boldsymbol{\beta}\partial \alpha_l}\right);$$
$$\left(\frac{\partial \hat{\boldsymbol{\mu}}_i}{\partial \alpha_l}\right)' = \left(\frac{\partial g^{-1}\left(\hat{\mathbf{X}}_{i1}'\boldsymbol{\beta}\right)}{\partial \alpha_l}, \dots, \frac{\partial g^{-1}\left(\hat{\mathbf{X}}_{im_i}'\boldsymbol{\beta}\right)}{\partial \alpha_l}\right)$$

for $l = 1, \dots, \dim(\boldsymbol{\alpha})$. The quantities can be computed using the chain rule. Assuming a linear measurement error model

$$E[c_i(t_{ik})|C_i(t_{ik}), t_{ik}, \mathbf{W}_i(t_{ik})] = \alpha_0 + \alpha_1 C_i(t_{ik}) + \alpha_2 t_{ik} + \alpha_3 C_i(t_{ik})t_{ik} + \boldsymbol{\alpha}_4'\mathbf{W}_i(t_{ik}),$$

the cumulative average exposure is

$$\hat{s}_i(t_{ij}) = \frac{\sum_{k=1}^{j-1}(t_{i(k+1)} - t_{ik})\hat{c}_i(t_{ik})}{t_{ij} - t_{i1}}$$

for $j = 2, \dots, m_i$ and $\hat{s}_i(t_{i1}) = \hat{c}_i(t_{i1})$, $i = 1, \dots, n_1$. In such cases,

$$\frac{\partial \hat{\mathbf{X}}_{ij}'\boldsymbol{\beta}}{\partial \alpha_l} = 0 + \beta_1\frac{\partial \hat{s}_i(t_{ij})}{\partial \alpha_l} + 0 + \beta_3\frac{\partial \hat{s}_i(t_{ij})t_{ij}}{\partial \alpha_l} + 0$$

for $l = 0, \dots, 4$ and $j = 1, \dots, m_i$, where

$$\frac{\partial \hat{s}_i(t_{ij})}{\partial \alpha_0} = 1;$$
$$\frac{\partial \hat{s}_i(t_{ij})}{\partial \alpha_1} = Z_i(t_{ij});$$
$$\frac{\partial \hat{s}_i(t_{i1})}{\partial \alpha_2} = t_{i1},\ \frac{\partial \hat{s}_i(t_{ij})}{\partial \alpha_2} = \frac{\sum_{k=1}^{j-1}(t_{i(k+1)} - t_{ik})t_{ik}}{t_{ij} - t_{i1}} \text{ for } j = 2, \dots, m_i;$$
$$\frac{\partial \hat{s}_i(t_{i1})}{\partial \alpha_3} = C_i(t_{i1})t_{i1},\ \frac{\partial \hat{s}_i(t_{ij})}{\partial \alpha_3} = \frac{\sum_{k=1}^{j-1}(t_{i(k+1)} - t_{ik})C_i(t_{ik})t_{ik}}{t_{ij} - t_{i1}} \text{ for } j = 2, \dots, m_i;$$
$$\frac{\partial \hat{s}_i(t_{i1})}{\partial \boldsymbol{\alpha}_4} = \mathbf{W}_i'(t_{i1}),\ \frac{\partial \hat{s}_i(t_{ij})}{\partial \boldsymbol{\alpha}_4} = \frac{\sum_{k=1}^{j-1}(t_{i(k+1)} - t_{ik})\mathbf{W}_i'(t_{ik})}{t_{ij} - t_{i1}} \text{ for } j = 2, \dots, m_i.$$

Note that if the true exposure is used for those individuals belong to the IVS, then the derivatives above will differ as $c_i(t_{ij})$ will not be estimated as $\hat{c}_i(t_{ij})$ for $i = n_1+1, \dots, n_1+n_2$ and $j = 1, \dots, m_i$. For example, if the true exposure was available at $j = 2$ for individual i, then $\hat{s}_i(t_{ij})$ was constructed from $\hat{c}_i(t_{i1}), c_i(t_{i2}), \hat{c}_i(t_{i3}), \dots, \hat{c}_i(t_{im_i})$. Hence, we should modify the derivatives with respect to $\boldsymbol{\alpha}$ accordingly, for example,

$$\frac{\partial \hat{s}_i(t_{i1})}{\partial \alpha_0} = 1,\ \frac{\partial \hat{s}_i(t_{i2})}{\partial \alpha_0} = 1,\ \frac{\partial \hat{s}_i(t_{i3})}{\partial \alpha_0} = \frac{1}{2},\ \frac{\partial \hat{s}_i(t_{i4})}{\partial \alpha_0} = \frac{2}{3},\ \frac{\partial \hat{s}_i(t_{i5})}{\partial \alpha_0} = \frac{3}{4},$$

assuming that $m_i = 5$.

Finally, the variance estimates of the inverse variance weighted estimator can be computed from $\widehat{\text{Var}}(\hat{\boldsymbol{\beta}}_M)$ and $\widehat{\text{Var}}(\hat{\boldsymbol{\beta}}_I)$. While the former is the variance estimate of the estimator following from the MS analyses as shown above, the latter is the variance estimate of the maximum likelihood estimator following from a generalized linear model based on the IVS data alone,

$$\widehat{\text{Var}}(\hat{\boldsymbol{\beta}}_I) = \left[- \sum_{i=n_1+1}^{n_1+n_2} \frac{\partial^2 \mathbf{L}_i(\boldsymbol{\beta})}{\partial \boldsymbol{\beta} \partial \boldsymbol{\beta}'} \right]^{-1} |_{\boldsymbol{\beta}=\hat{\boldsymbol{\beta}}}.$$

For example, should we consider binary response, then the resulting variance estimate follows from the inverse information matrix of a standard logistic regression evaluated at the maximum likelihood estimate.

A2. Multiple validated exposure measurements in the validation study

As the Ordinary Least Square (OLS) regression generalizes to the generalized estimating equation (GEE), it is straightforward to modify estimates of $\mathbf{A}(\boldsymbol{\theta})$ and $\mathbf{B}(\boldsymbol{\theta})$ accordingly. For example, assuming a linear measurement error model, we now have

$$\text{Var}(\boldsymbol{\psi}_{\boldsymbol{\alpha}}) = \sum_{i=n_1+1}^{n_1+n_2} \left[\mathbf{C}_i \mathbf{V}_i^{-1} \left(\mathbf{c}_i - \mathbf{C}_i'\boldsymbol{\alpha}\right)\right] \left[\mathbf{C}_i \mathbf{V}_i^{-1} \left(\mathbf{c}_i - \mathbf{C}_i'\boldsymbol{\alpha}\right)\right]' |_{\boldsymbol{\alpha}=\hat{\boldsymbol{\alpha}}}$$

and for the MS/IVS design,

$$\text{Cov}(\boldsymbol{\psi}_{\boldsymbol{\alpha}}, \boldsymbol{\psi}_{\boldsymbol{\beta}}) \approx \sum_{i=n_1+1}^{n_1+n_2} \left[\mathbf{C}_i \mathbf{V}_i^{-1} \left(\mathbf{c}_i - \mathbf{C}_i'\boldsymbol{\alpha}\right)\right] \left[\left(\frac{\partial \boldsymbol{\mu}_i}{\partial \boldsymbol{\beta}}\right)' \boldsymbol{\Sigma}_i^{-1}(\mathbf{Y}_i - \boldsymbol{\mu}_i) \right]' |_{\boldsymbol{\mu}_i=\hat{\boldsymbol{\mu}}_i, \boldsymbol{\alpha}=\hat{\boldsymbol{\alpha}}}.$$

Moreover,

$$E\left(\frac{\partial \boldsymbol{\psi}_{\boldsymbol{\alpha}}}{\partial \boldsymbol{\alpha}}\right) = - \sum_{i=n_1+1}^{n_1+n_2} \mathbf{C}_i \mathbf{V}_i^{-1} \mathbf{C}_i'.$$

If the true exposure are used for those individuals belong to the IVS under the MS/IVS design, then no imputation occurs for $i = n_1+1, \ldots, n_1+n_2$ at $j = 1, \ldots, v_i$. In particular, if the true exposure are available at all time points in the IVS, i.e., $v_i = m_i$ for $i = n_1+1, \ldots, n_1+n_2$, we have

$$\begin{aligned} E\left(\frac{\partial \boldsymbol{\psi}_{\boldsymbol{\beta}}}{\partial \alpha_l}\right) &= \sum_{i=1}^{n_1} \left[\left(\frac{\partial \boldsymbol{\mu}_i}{\partial \boldsymbol{\beta} \partial \alpha_l}\right)' \boldsymbol{\Sigma}_i^{-1}(\mathbf{Y}_i - \boldsymbol{\mu}_i) - \left(\frac{\partial \boldsymbol{\mu}_i}{\partial \boldsymbol{\beta}}\right)' \boldsymbol{\Sigma}_i^{-1} \frac{\partial \boldsymbol{\mu}_i}{\partial \alpha_l} \right] |_{\boldsymbol{\mu}_i=\hat{\boldsymbol{\mu}}_i} \\ &= - \sum_{i=1}^{n_1} \left[\left(\frac{\partial \boldsymbol{\mu}_i}{\partial \boldsymbol{\beta}}\right)' \boldsymbol{\Sigma}_i^{-1} \frac{\partial \boldsymbol{\mu}_i}{\partial \alpha_l} \right] |_{\boldsymbol{\mu}_i=\hat{\boldsymbol{\mu}}_i}. \end{aligned}$$

As for the inverse variance weighted estimator, the variance estimates can be computed from $\widehat{\text{Var}}(\hat{\boldsymbol{\beta}}_M)$ and $\widehat{\text{Var}}(\hat{\boldsymbol{\beta}}_I)$, which are the variance estimates of the estimators following from the MS analyses as shown above and a standard GEE analysis based on the IVS data alone, i.e.,

$$\widehat{\text{Var}}(\hat{\boldsymbol{\beta}}_I) = \left[\sum_{i=n_1+1}^{n_1+n_2} \frac{\partial \mathbf{U}_i(\boldsymbol{\beta})}{\partial \boldsymbol{\beta}} \right]^{-1} \left[\sum_{i=n_1+1}^{n_1+n_2} \mathbf{U}_i(\boldsymbol{\beta}) \mathbf{U}_i(\boldsymbol{\beta})' \right] \left[\sum_{i=n_1+1}^{n_1+n_2} \frac{\partial \mathbf{U}_i(\boldsymbol{\beta})}{\partial \boldsymbol{\beta}} \right]^{-1'} |_{\boldsymbol{\beta}=\hat{\boldsymbol{\beta}}},$$

respectively.

A3. Robustness of the exposure measurement error correction procedure

We hereby show that, if the main study model requires a time by exposure interaction term, the measurement error model also requires this. Otherwise, biased estimates will be obtained as in the simulation studies of Section 3.3. Suppose that the exposure-time interaction term under the linear measurement error model,

$$E[c_i(t_{ik})|C_i(t_{ik}), t_{ik}, \mathbf{W}_i(t_{ik})] = \alpha_0 + \alpha_1 C_i(t_{ik}) + \alpha_2 t_{ik} + \alpha_3 C_i(t_{ik}) t_{ik} + \boldsymbol{\alpha}_4' \mathbf{W}_i(t_{ik}),$$

is omitted so that we have the misspecified model

$$E[c_i(t_{ik})|C_i(t_{ik}), t_{ik}, \mathbf{W}_i(t_{ik})] = \alpha_0 + \tilde{\alpha}_1 C_i(t_{ik}) + \tilde{\alpha}_2 t_{ik} + \boldsymbol{\alpha}_4' \mathbf{W}_i(t_{ik}).$$

As a result, if interest lies in the cumulative average exposure, the outcome model becomes

$$\begin{aligned}
E[Y_i(t_{ij})|\tilde{C}_i(t_{ij}), \tilde{t}_{ij}, \tilde{\mathbf{W}}_i(t_{ij})] \approx\ & g^{-1}\left(\beta_0 + E\left[h(\tilde{c}_i(t_{ij}))|\tilde{C}_i(t_{ij}), \tilde{t}_{ij}, \tilde{\mathbf{W}}_i(t_{ij})\right]' \beta_1 + \beta_2 t_{ij}\right. \\
& \left. + E\left[h(\tilde{c}_i(t_{ij}))|\tilde{C}_i(t_{ij}), \tilde{t}_{ij}, \tilde{\mathbf{W}}_i(t_{ij})\right]' \beta_3 t_{ij} + \tilde{\mathbf{W}}_i'(t_{ij})\boldsymbol{\beta}_4\right) \\
=\ & g^{-1}\left(\beta_0 + \frac{\sum_{k=1}^{j-1}(t_{i(k+1)} - t_{ik}) E\left[c_i(t_{ik})|C_i(t_{ik}), t_{ik}, \mathbf{W}_i(t_{ik})\right]}{t_{ij} - t_{i1}} \beta_1 + \beta_2 t_{ij}\right. \\
& \left. + \frac{\sum_{k=1}^{j-1}(t_{i(k+1)} - t_{ik}) E\left[c_i(t_{ik})|C_i(t_{ik}), t_{ik}, \mathbf{W}_i(t_{ik})\right]}{t_{ij} - t_{i1}} \beta_3 t_{ij} + \tilde{\mathbf{W}}_i'(t_{ij})\boldsymbol{\beta}_4\right) \\
=\ & g^{-1}\left(\beta_0 + \frac{\sum_{k=1}^{j-1}(t_{i(k+1)} - t_{ik})[\alpha_0 + \tilde{\alpha}_1 C_i(t_{ik}) + \tilde{\alpha}_2 t_{ik} + \boldsymbol{\alpha}_4' \mathbf{W}_i(t_{ik})]}{t_{ij} - t_{i1}} \beta_1 + \beta_2 t_{ij}\right. \\
& \left. + \frac{\sum_{k=1}^{j-1}(t_{i(k+1)} - t_{ik})[\alpha_0 + \tilde{\alpha}_1 C_i(t_{ik}) + \tilde{\alpha}_2 t_{ik} + \boldsymbol{\alpha}_4' \mathbf{W}_i(t_{ik})]}{t_{ij} - t_{i1}} \beta_3 t_{ij} + \tilde{\mathbf{W}}_i'(t_{ij})\boldsymbol{\beta}_4\right)
\end{aligned}$$

instead of the approximation of the correctly specified conditional expectation,

$$\begin{aligned}
g^{-1}\left(\beta_0 + \frac{\sum_{k=1}^{j-1}(t_{i(k+1)} - t_{ik})[\alpha_0 + \alpha_1 C_i(t_{ik}) + \alpha_2 t_{ik} + \alpha_3 C_i(t_{ik}) t_{ik} + \boldsymbol{\alpha}_4' \mathbf{W}_i(t_{ik})]}{t_{ij} - t_{i1}} \beta_1 + \beta_2 t_{ij}\right. \\
\left. + \frac{\sum_{k=1}^{j-1}(t_{i(k+1)} - t_{ik})[\alpha_0 + \alpha_1 C_i(t_{ik}) + \alpha_2 t_{ik} + \alpha_3 C_i(t_{ik}) t_{ik} + \boldsymbol{\alpha}_4' \mathbf{W}_i(t_{ik})]}{t_{ij} - t_{i1}} \beta_3 t_{ij} + \tilde{\mathbf{W}}_i'(t_{ij})\boldsymbol{\beta}_4\right).
\end{aligned}$$

Take the standard logistic regression model for example, where $g(\cdot)$ is the logit function, $g(x) = \log x/(1-x)$. The difference between the two log odds presented above is typically not zero. Thus, the conditional expectation following from the misspecified measurement error model differs from the one following from the correctly specified measurement error model, and hence, will lead to a biased generalized estimating equation when substituting into (2.3). While consistent estimators of β_1 and β_3 will not be obtained, the asymptotic bias will depend on the measurement error model, the exposure history function of interest, and the link function of the outcome model.

A4. Further notes on time scale of analysis

We hereby show that the time scale used in the measurement error model does not have to be the same as that in the outcome model for analysis. For jth assessment of participant i, let t_{ij} and t'_{ij} denote time since baseline assessment and age, respectively. Suppose that time since baseline is used as the time scale in the outcome model and age is used in the measurement error model. In such cases, baseline age, t'_{i1}, should be considered in the outcome model as a potential confounder. Let $\mathbf{W}_i(t_{ij})_{-t'_{i1}}$ denote potential confounders excluding t'_{i1}. It follows that

$$
\begin{aligned}
E[Y_i(t_{ij})|\tilde{C}_i(t_{ij}), \tilde{t}_{ij}, \tilde{\mathbf{W}}_i(t_{ij})] \approx g^{-1} \Bigg(& \beta_0 + E\left[h(\tilde{c}_i(t_{ij}))|\tilde{C}_i(t_{ij}), \tilde{t}_{ij}, t'_{i1}, \tilde{\mathbf{W}}_i(t_{ij})_{-t'_{i1}}\right]' \beta_1 + \beta_2 t_{ij} \\
& + E\left[h(\tilde{c}_i(t_{ij}))|\tilde{C}_i(t_{ij}), \tilde{t}_{ij}, t'_{i1}, \tilde{\mathbf{W}}_i(t_{ij})_{-t'_{i1}}\right]' \beta_3 t_{ij} + \tilde{\mathbf{W}}'_i(t_{ij})\boldsymbol{\beta}_4 \Bigg),
\end{aligned}
$$

where

$$
E\left[h(\tilde{c}_i(t_{ij}))|\tilde{C}_i(t_{ij}), \tilde{t}_{ij}, t'_{i1}, \tilde{\mathbf{W}}_i(t_{ij})_{-t'_{i1}}\right] = \frac{\sum_{k=1}^{j-1}(t_{i(k+1)} - t_{ik}) E\left[c_i(t_{ik})|\tilde{C}_i(t_{ij}), \tilde{t}_{ij}, t'_{i1}, \tilde{\mathbf{W}}_i(t_{ij})_{-t'_{i1}}\right]}{t_{ij} - t_{i1}}.
$$

Note the bijective transformation between t'_{ik} and t_{ik} given t'_{i1}, $t'_{ik} = t_{ik} + t'_{i1}$. We can write $c_i(t_{ik})$, $C_i(t_{ik})$, and $\mathbf{W}_i(t_{ik})$ as $c_i(t'_{ik})$, $C_i(t'_{ik})$, and $\mathbf{W}_i(t'_{ik})$, respectively. Therefore,

$$
\begin{aligned}
E\left[h(\tilde{c}_i(t_{ij}))|\tilde{C}_i(t_{ij}), \tilde{t}_{ij}, t'_{i1}, \tilde{\mathbf{W}}_i(t_{ij})_{-t'_{i1}}\right] &= \frac{\sum_{k=1}^{j-1}(t_{i(k+1)} - t_{ik}) E\left[c_i(t'_{ik})|\tilde{C}_i(t'_{ij}), \tilde{t}'_{ij}, \tilde{\mathbf{W}}_i(t'_{ij})_{-t'_{i1}}\right]}{t_{ij} - t_{i1}} \\
&= \frac{\sum_{k=1}^{j-1}(t_{i(k+1)} - t_{ik}) E\left[c_i(t'_{ik})|C_i(t'_{ik}), t'_{ik}, \mathbf{W}_i(t'_{ik})_{-t'_{i1}}\right]}{t_{ij} - t_{i1}}
\end{aligned}
$$

assuming the localized error assumption. Hence,

$$
\begin{aligned}
E[Y_i(t_{ij})|\tilde{C}_i(t_{ij}), \tilde{t}_{ij}, \tilde{\mathbf{W}}_i(t_{ij})] \approx g^{-1} \Bigg(& \beta_0 + \frac{\sum_{k=1}^{j-1}(t_{i(k+1)} - t_{ik}) E\left[c_i(t'_{ik})|C_i(t'_{ik}), t'_{ik}, \mathbf{W}_i(t'_{ik})_{-t'_{i1}}\right]}{t_{ij} - t_{i1}} \beta_1 + \beta_2 t_{ij} \\
& + \frac{\sum_{k=1}^{j-1}(t_{i(k+1)} - t_{ik}) E\left[c_i(t'_{ik})|C_i(t'_{ik}), t'_{ik}, \mathbf{W}_i(t'_{ik})_{-t'_{i1}}\right]}{t_{ij} - t_{i1}} \beta_3 t_{ij} + \tilde{\mathbf{W}}'_i(t_{ij})\boldsymbol{\beta}_4 \Bigg)
\end{aligned}
$$

so that time since the baseline assessment is used in the outcome model, whereas age is used in the measurement error model. Allowing for distinct time scales in the outcome and measurement error models grants more flexibility in applying our proposed method under main study/validation study designs.

Appendix B. Additional tables for empirical studies

Table 1: Relative biases (RBias), average standard errors (ASE), empirical standard errors (ESE), and empirical coverage probabilities (CPs) of the 95% confidence intervals of the estimator $\hat{\beta}_3$ following the proposed method and the uncorrected analyses under the MS/EVS design. The true exposure was available at all five time points in the validation study. The working correlation matrix was specified as AR(1) in the GEE analyses. $n_1 = 5000/2000$, $n_2 = 500/200$, and $(\beta_1, \beta_3) = (\log 1.2, -\log 1.1)/(\log 1.2, -\log 1.5)$. The correlation between the true and surrogate exposure was either 0.90 or 0.75.

		Uncorrected				Proposed			
β_3	Cor(c, C)	RBias	ASE	ESE	CP	RBias	ASE	ESE	CP
		$n_1 = 5000, n_2 = 500$							
$-\log 1.1$	0.90	24.21%	0.015	0.015	0.64	-0.37%	0.014	0.013	0.96
	0.75	24.11%	0.015	0.015	0.65	-0.61%	0.014	0.013	0.96
$-\log 1.5$	0.90	26.17%	0.023	0.024	0.01	-1.12%	0.029	0.023	0.99
	0.75	23.15%	0.023	0.024	0.03	-5.12%	0.029	0.024	0.93
		$n_1 = 5000, n_2 = 200$							
$-\log 1.1$	0.90	24.83%	0.015	0.015	0.63	-0.31%	0.014	0.013	0.96
	0.75	24.57%	0.015	0.015	0.66	-0.77%	0.014	0.013	0.96
$-\log 1.5$	0.90	26.14%	0.023	0.022	0.04	-1.36%	0.029	0.022	0.98
	0.75	23.05%	0.023	0.022	0.01	-5.43%	0.030	0.023	0.94
		$n_1 = 2000, n_2 = 500$							
$-\log 1.1$	0.90	26.10%	0.024	0.024	0.80	0.64%	0.022	0.021	0.95
	0.75	25.63%	0.024	0.024	0.82	0.15%	0.022	0.022	0.95
$-\log 1.5$	0.90	26.10%	0.037	0.037	0.14	-0.40%	0.046	0.037	0.97
	0.75	23.55%	0.036	0.037	0.25	-4.51%	0.046	0.038	0.96
		$n_1 = 2000, n_2 = 200$							
$-\log 1.1$	0.90	25.07%	0.024	0.023	0.83	0.05%	0.022	0.021	0.96
	0.75	24.94%	0.024	0.023	0.83	-0.29%	0.022	0.021	0.96
$-\log 1.5$	0.90	25.67%	0.036	0.034	0.17	-1.95%	0.046	0.034	0.99
	0.75	23.08%	0.036	0.034	0.26	-5.60%	0.046	0.034	0.98

For a given MS sample size, relative biases improved as the validation study sample size increased. In some cases, this was not observed for the estimate of the parameter of interest, β_3, but observed for the estimate of the parameter corresponding to the main effect of the cumulative average exposure, β_1.

Table 2: Relative biases (RBias), average standard errors (ASE), empirical standard errors (ESE), and empirical coverage probabilities (CPs) of the 95% confidence intervals of the estimator $\hat{\beta}_3$ following the proposed method and the uncorrected analyses under the MS/IVS design. The true exposure was available at all five time points in the validation study. The working correlation matrix was specified as AR(1) in the GEE analyses. $n_1 = 5000/2000$, $n_2 = 500/200$, and $(\beta_1, \beta_3) = (\log 1.2, -\log 1.1)/(\log 1.2, -\log 1.5)$. The correlation between the true and surrogate exposure was either 0.90 or 0.75.

		Uncorrected				Proposed				Proposed - True				Proposed - Inv			
β_3	Cor(c, C)	RBias	ASE	ESE	CP	RBias	ASE	ESE	CP	RBias	ASE	ESE	CP	RBias	ASE	ESE	CP
						$n_1 = 5000, n_2 = 500$											
$-\log 1.1$	0.90	24.82%	0.014	0.015	0.63	0.28%	0.013	0.012	0.97	0.28%	0.013	0.012	0.97	0.20%	0.013	0.012	0.97
	0.75	24.74%	0.014	0.015	0.63	0.06%	0.013	0.012	0.96	0.12%	0.013	0.012	0.97	0.00%	0.013	0.012	0.97
$-\log 1.5$	0.90	26.05%	0.022	0.022	0.00	-1.06%	0.028	0.022	0.98	-0.95%	0.027	0.022	0.98	-0.87%	0.027	0.022	0.97
	0.75	23.08%	0.022	0.022	0.01	-5.13%	0.028	0.022	0.94	-4.65%	0.027	0.022	0.95	-4.17%	0.027	0.022	0.94
						$n_1 = 5000, n_2 = 200$											
$-\log 1.1$	0.90	24.33%	0.015	0.014	0.66	-0.60%	0.014	0.012	0.98	-0.65%	0.014	0.012	0.97	-0.71%	0.014	0.012	0.97
	0.75	24.36%	0.015	0.014	0.68	-0.99%	0.014	0.013	0.97	-1.07%	0.014	0.013	0.97	-1.14%	0.014	0.013	0.97
$-\log 1.5$	0.90	26.00%	0.023	0.023	0.00	-1.47%	0.029	0.023	0.99	-1.44%	0.029	0.023	0.99	-1.40%	0.028	0.023	0.99
	0.75	23.11%	0.022	0.023	0.02	-5.39%	0.030	0.023	0.95	-5.26%	0.029	0.022	0.95	-4.97%	0.029	0.023	0.95
						$n_1 = 2000, n_2 = 500$											
$-\log 1.1$	0.90	25.96%	0.021	0.021	0.79	1.24%	0.020	0.019	0.97	1.41%	0.020	0.019	0.96	1.45%	0.019	0.019	0.96
	0.75	25.82%	0.021	0.021	0.79	0.94%	0.020	0.019	0.97	1.29%	0.019	0.019	0.96	1.31%	0.019	0.019	0.95
$-\log 1.5$	0.90	26.52%	0.033	0.032	0.09	-0.62%	0.041	0.032	0.98	-0.34%	0.041	0.032	0.98	-0.03%	0.038	0.033	0.98
	0.75	23.71%	0.032	0.031	0.15	-4.49%	0.041	0.031	0.97	-3.63%	0.040	0.030	0.98	-2.49%	0.037	0.031	0.97
						$n_1 = 2000, n_2 = 200$											
$-\log 1.1$	0.90	25.70%	0.023	0.023	0.80	0.38%	0.021	0.020	0.97	0.52%	0.021	0.020	0.97	0.81%	0.021	0.020	0.96
	0.75	25.57%	0.023	0.022	0.81	0.09%	0.021	0.020	0.96	0.40%	0.021	0.019	0.96	0.70%	0.021	0.020	0.96
$-\log 1.5$	0.90	26.16%	0.035	0.036	0.14	-1.31%	0.043	0.037	0.98	-1.14%	0.043	0.037	0.98	-0.84%	0.042	0.038	0.97
	0.75	23.49%	0.034	0.036	0.20	-5.00%	0.044	0.037	0.96	-4.55%	0.043	0.036	0.97	-3.74%	0.042	0.038	0.96

"Proposed" refers to the method which uses measurement error corrected exposures for all participants; "Proposed - True" refers to the method which uses true exposures whenever available in the IVS; "Proposed - Inv" refers to the method which leads to an inverse-variance weighted estimator.

Table 3: Relative biases (RBias), average standard errors (ASE), empirical standard errors (ESE), and empirical coverage probabilities (CPs) of the 95% confidence intervals of the estimator $\hat{\beta}_3$ following the proposed method and the uncorrected analyses under the MS/EVS and MS/IVS designs. Only one measurement of the true exposure was available in the validation study. The measurement error model was misspecified by omitting the interaction term. The working correlation matrix was specified as AR(1) in the GEE analyses. $n_1 = 5000$, $n_2 = 500$, and $(\beta_1, \beta_3) = (\log 1.2, -\log 1.1)/(\log 1.2, -\log 1.5)$. The correlation between the true and surrogate exposure was either 0.90 or 0.75.

			Uncorrected				Proposed			
Design	β_3	Cor(c, C)	RBias	ASE	ESE	CP	RBias	ASE	ESE	CP
EVS	$-\log 1.1$	0.90	23.44%	0.015	0.015	0.66	-22.98%	0.010	0.009	0.37
		0.75	23.36%	0.015	0.015	0.65	-23.06%	0.010	0.009	0.39
	$-\log 1.5$	0.90	26.16%	0.023	0.021	0.00	-18.76%	0.022	0.015	0.03
		0.75	23.59%	0.023	0.021	0.01	-20.80%	0.023	0.017	0.02
IVS	$-\log 1.1$	0.90	24.08%	0.014	0.016	0.63	-22.63%	0.009	0.010	0.37
		0.75	23.90%	0.014	0.016	0.64	-22.72%	0.010	0.010	0.38
	$-\log 1.5$	0.90	26.17%	0.022	0.023	0.00	-18.77%	0.021	0.017	0.03
		0.75	23.30%	0.022	0.022	0.01	-21.01%	0.022	0.019	0.02
IVS True	$-\log 1.1$	0.90	24.08%	0.014	0.016	0.63	-22.42%	0.009	0.010	0.36
		0.75	23.90%	0.014	0.016	0.64	-22.47%	0.010	0.010	0.40
	$-\log 1.5$	0.90	26.17%	0.022	0.023	0.00	-18.52%	0.021	0.017	0.02
		0.75	23.30%	0.022	0.022	0.01	-20.71%	0.022	0.019	0.02
IVS Inv	$-\log 1.1$	0.90	24.08%	0.014	0.016	0.63	-24.32%	0.010	0.010	0.33
		0.75	23.90%	0.014	0.016	0.64	-24.97%	0.010	0.010	0.34
	$-\log 1.5$	0.90	26.17%	0.022	0.023	0.00	-22.90%	0.021	0.016	0.00
		0.75	23.30%	0.022	0.022	0.01	-26.98%	0.022	0.017	0.00

Under the MS/IVS design, "IVS" refers to the method which uses measurement error corrected exposures for all participants; "IVS True" refers to the method which uses true exposures whenever available in the IVS; "IVS Inv" refers to the method which leads to an inverse-variance weighted estimator.

Table 4: Relative biases (RBias), average standard errors (ASE), empirical standard errors (ESE), and empirical coverage probabilities (CPs) of the 95% confidence intervals of the estimator $\hat{\beta}_3$ following the proposed method and the uncorrected analyses under the MS/EVS and MS/IVS designs. Only one measurement of the true exposure was available in the validation study. The working correlation matrix was specified as exchangeable and independence in the GEE analyses of both main and validation studies. $n_1 = 5000$, $n_2 = 500$, and $(\beta_1, \beta_3) = (\log 1.2, -\log 1.1)/(\log 1.2, -\log 1.5)$. The correlation between the true and surrogate exposure was either 0.90 or 0.75.

			Uncorrected				Proposed			
Design	β_3	Cor(c, C)	RBias	ASE	ESE	CP	RBias	ASE	ESE	CP
			Exchangeable correlation structure							
EVS	$-\log 1.1$	0.90	22.89%	0.015	0.015	0.66	-1.29%	0.014	0.013	0.96
		0.75	22.80%	0.015	0.015	0.66	-1.63%	0.014	0.014	0.94
	$-\log 1.5$	0.90	26.01%	0.023	0.021	0.00	-0.98%	0.030	0.021	0.99
		0.75	23.41%	0.023	0.021	0.01	-4.58%	0.032	0.023	0.97
IVS	$-\log 1.1$	0.90	23.54%	0.014	0.016	0.64	-0.77%	0.013	0.014	0.95
		0.75	23.37%	0.014	0.016	0.66	-1.16%	0.014	0.014	0.96
	$-\log 1.5$	0.90	26.02%	0.022	0.023	0.00	-0.89%	0.028	0.023	0.98
		0.75	23.13%	0.022	0.022	0.01	-4.77%	0.031	0.026	0.93
IVS True	$-\log 1.1$	0.90	23.54%	0.014	0.016	0.64	-0.74%	0.013	0.014	0.95
		0.75	23.37%	0.014	0.016	0.66	-1.10%	0.014	0.014	0.95
	$-\log 1.5$	0.90	26.02%	0.022	0.023	0.00	-0.88%	0.028	0.023	0.98
		0.75	23.13%	0.022	0.022	0.01	-4.70%	0.031	0.026	0.92
IVS Inv	$-\log 1.1$	0.90	23.54%	0.014	0.016	0.64	-5.15%	0.014	0.014	0.93
		0.75	23.37%	0.014	0.016	0.66	-6.84%	0.014	0.013	0.93
	$-\log 1.5$	0.90	26.02%	0.022	0.023	0.00	-10.11%	0.028	0.023	0.72
		0.75	23.13%	0.022	0.022	0.01	-17.95%	0.030	0.026	0.29
			Independence correlation structure							
EVS	$-\log 1.1$	0.90	23.37%	0.015	0.015	0.66	-1.24%	0.014	0.013	0.96
		0.75	23.28%	0.015	0.015	0.65	-1.59%	0.014	0.014	0.94
	$-\log 1.5$	0.90	26.26%	0.023	0.021	0.00	-0.98%	0.030	0.021	0.99
		0.75	23.71%	0.023	0.021	0.00	-4.59%	0.032	0.023	0.97
IVS	$-\log 1.1$	0.90	24.04%	0.014	0.016	0.63	-0.71%	0.013	0.014	0.95
		0.75	23.86%	0.014	0.016	0.64	-1.09%	0.014	0.014	0.96
	$-\log 1.5$	0.90	26.28%	0.022	0.023	0.00	-0.89%	0.028	0.023	0.98
		0.75	23.43%	0.022	0.022	0.01	-4.78%	0.031	0.026	0.93
IVS True	$-\log 1.1$	0.90	24.04%	0.014	0.016	0.63	-0.68%	0.013	0.014	0.95
		0.75	23.86%	0.014	0.016	0.64	-1.03%	0.014	0.014	0.95
	$-\log 1.5$	0.90	26.28%	0.022	0.023	0.00	-0.87%	0.028	0.023	0.98
		0.75	23.43%	0.022	0.022	0.01	-4.70%	0.031	0.026	0.92
IVS Inv	$-\log 1.1$	0.90	24.04%	0.014	0.016	0.63	-5.09%	0.014	0.014	0.94
		0.75	23.86%	0.014	0.014	0.64	-6.78%	0.014	0.014	0.93
	$-\log 1.5$	0.90	26.28%	0.022	0.023	0.00	-10.11%	0.028	0.023	0.72
		0.75	23.43%	0.022	0.022	0.01	-17.97%	0.030	0.026	0.29

Under the MS/IVS design, "IVS" refers to the method which uses measurement error corrected exposures for all participants; "IVS True" refers to the method which uses true exposures whenever available in the IVS; "IVS Inv" refers to the method which leads to an inverse-variance weighted estimator.

Table 5: Relative biases (RBias), average standard errors (ASE), empirical standard errors (ESE), and empirical coverage probabilities (CPs) of the 95% confidence intervals of the estimator $\hat{\beta}_3$ following the proposed method and the uncorrected analyses under the MS/EVS and MS/IVS designs. The working correlation matrix was specified as AR(1) in the GEE analyses. $n_1 = 5000$, $n_2 = 500$, and $(\beta_1, \beta_3) = (\log 1.2, -\log 2)$. The correlation between the true and surrogate exposure was 0.85, 0.80, or 0.75, corresponding to a $\boldsymbol{\beta}'\text{Var}(\mathbf{X}|\tilde{C}, \tilde{t}, \tilde{W})\boldsymbol{\beta}$ of 0.42, 0.63, and 0.88, respectively.

		Uncorrected				Proposed			
Design	$\text{Cor}(c, C)$	RBias	ASE	ESE	CP	RBias	ASE	ESE	CP
EVS	0.85	15.00%	0.031	0.030	0.08	-6.43%	0.044	0.033	0.91
	0.80	12.14%	0.031	0.030	0.21	-9.98%	0.044	0.034	0.70
	0.75	9.11%	0.031	0.031	0.46	-13.65%	0.044	0.035	0.40
IVS	0.85	15.05%	0.030	0.030	0.05	-5.92%	0.043	0.031	0.91
	0.80	12.36%	0.030	0.029	0.17	-9.36%	0.042	0.031	0.72
	0.75	9.32%	0.029	0.028	0.38	-13.11%	0.042	0.031	0.42
IVS True	0.85	15.05%	0.030	0.030	0.05	-5.45%	0.042	0.031	0.92
	0.80	12.36%	0.030	0.029	0.17	-8.66%	0.042	0.031	0.76
	0.75	9.32%	0.029	0.028	0.38	-12.21%	0.041	0.030	0.44
IVS Inv	0.85	15.05%	0.030	0.030	0.05	-4.99%	0.041	0.032	0.91
	0.80	12.36%	0.030	0.029	0.17	-7.75%	0.040	0.032	0.79
	0.75	9.32%	0.029	0.028	0.38	-10.81%	0.040	0.032	0.55

Under the MS/IVS design, "IVS" refers to the method which uses measurement error corrected exposures for all participants; "IVS True" refers to the method which uses true exposures whenever available in the IVS; "IVS Inv" refers to the method which leads to an inverse-variance weighted estimator.

Appendix C. Additional information about main and validation study data

Table 1: Basic characteristics of the external validation study (274 participants and 426 person-months). Study data contains $PM_{2.5}$ monthly exposure measurements from panel studies between 1999 and 2002, the Multi-Ethnic Study of Atherosclerosis (MESA) study, and the Relationships of Indoor, Outdoor, and Personal Air (RIOPA) study.

Characteristic	Mean	Standard deviation	Minimum	Maximum
Monthly personal $PM_{2.5}$ of ambient origin ($\mu g/m^3$)	11.2	5.4	2.6	33.4
Monthly spatial-temporal predicted $PM_{2.5}$ ($\mu g/m^3$)	16.2	4.6	6.6	31.7
Age (Years)	57.9	18.5	18.0	90.4
	Percents of participants			
Having single $PM_{2.5}$ exposure measurement (%)	62.4%			
Having multiple $PM_{2.5}$ exposure measurements (%)	37.6%			

Table 2: Basic characteristics of the main study (65158 participants and 781896 person-months). Study data contains $PM_{2.5}$ monthly exposure measurements for 12 months prior to the anxiety assessments in 1993 and 2005.

Characteristic	Mean	Standard deviation	Minimum	Maximum
Monthly spatial-temporal predicted $PM_{2.5}$ ($\mu g/m^3$)	13.0	2.9	2.3	25.0
Age (Years)	43.7	7.6	28.0	60.0
Median income (Dollars)	54353	22485	0.0	200001
Proportion of black (%)	0.05	0.10	0.0	1.0
	Percents of participants			
Incidence of anxiety disorders (%)	10.0%			
Married (%)	79.8%			
Husband education more than high school (%)	70.7%			
Returned questionnaires in June (%)	63.9%			
Returned questionnaires in July (%)	16.1%			
Returned questionnaires in August (%)	5.8%			
Returned questionnaires in September (%)	2.6%			
Returned questionnaires in October (%)	6.0%			
Returned questionnaires in November (%)	2.7%			
Returned questionnaires in December (%)	3.0%			

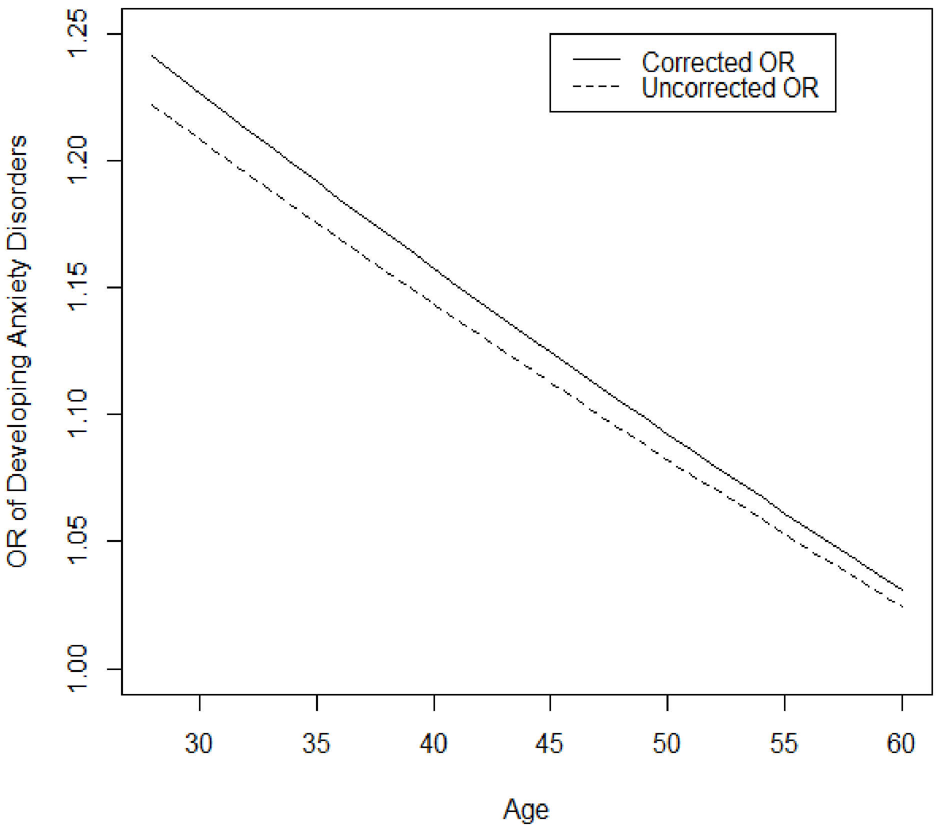

Figure 1: Measurement error corrected and uncorrected odds ratios of developing anxiety disorders per $10\mu g/m^3$ increment in prior 12-month moving average exposure to $PM_{2.5}$ in the NHS II. Anxiety was characterized by the Crown Crisp subscale score at the medical cut point of six. $PM_{2.5}$ chronic exposure was computed as the 12-month moving average exposure to $PM_{2.5}$ prior to anxiety assessments in 1993 and 2005.

References

[Liang and Zeger, 1986] Liang, K.-Y. and Zeger, S. L. (1986). Longitudinal data analysis using generalized linear models. *Biometrika*, 73(1):13–22.
